# Evaluation of the Returned Electromagnetic Signal from Retro-reflectors in Turbid Media

**DOI:** 10.1038/s41598-019-43059-z

**Published:** 2019-04-25

**Authors:** Iman Hassani nia, Skyler Wheaton, Hooman Mohseni

**Affiliations:** Department of Electrical Engineering and Computer Science, Northwestern University, Evanston, IL 60208 USA

**Keywords:** Applied optics, Optical physics

## Abstract

We provide first-principle theoretical and numerical simulations using the coherent Transfer Matrix Approach (TMA) to describe the behavior of the three main class of the optical beacons namely phase conjugators, reflectors, and retroreflectors within a turbid medium. Our theory describes the extraordinary enhancement (about 5 dB) offered by retroreflectors compared to reflectors in our detailed experiments and shows that they effectively act as local optical phase conjugators. Moreover, the performance of retroreflectors shows little degradation for increased light incident angles in turbid media, while the performance of reflectors degrades drastically. These results may find applications for detection of the echoes of electromagnetic radiation in turbid media.

## Introduction

Light is the main tool for non-invasive measurement of fundamental quantities of the universe. Control and manipulation of the light propagation in scattering tissue are quite influential for a broad range of measurements in life sciences, astronomy, and telecommunication. Almost all these applications share one common feature; They require highly confined temporal and spatial optical modes at the location of interest within the scattering media and fast scanning ability to boost the measurement fidelity. Consequently, novel ideas for addressing these issues have been proposed and implemented. These strategies rely on either non-linear excitation (as in multiphoton microscopy systems^1–6^), interferometric effects in homodyne/heterodyne detection systems^6–8^, time reversal based on phase conjugation^9–11^, speckle autocorrelation utilizing memory effect^12^, and techniques based on iterative wavefront shaping^13–17^. The nonlinear systems work on the principle of generation of enough optical intensity at the desired voxel to excite nonlinear emission that can be discriminated with high spatial resolution through wavelength filtration. Unfortunately, these systems fail for depths larger than about 5*l*, where *l* is the mean free scattering length, since almost 99% of light experiences multiple scattering^9^. Other approaches based on interference and phase conjugation have gained a significant attention for enhancing temporal and spatial resolution. While the Boltzmann picture of transport can describe light propagation in weakly disordered media, it fails to predict various coherent mechanisms encountered in phase conjugation and interference methods. In this letter, we scrutinize *coherent* optical back coupling from a scatterer inside the turbid media using generalized transmission matrix approach (TMA). In particular, we compare the performance of a retroreflector and a reflector. Our motivation is from an intuition that a retroreflector might perform better, since they return light backward through the same path of incidence, producing an effect similar to time reversal. While this work uses scattering parameters similar to those of human tissue, any other multiple scattering phenomena such as Anderson localization could also be included in our generalized approach. We further evaluate our theoretical model by numerical simulation, and experimental measurements. These results suggest that a retroreflector is similar to an optical phase conjugate mirror, but with important differences.

**Figure 1:**
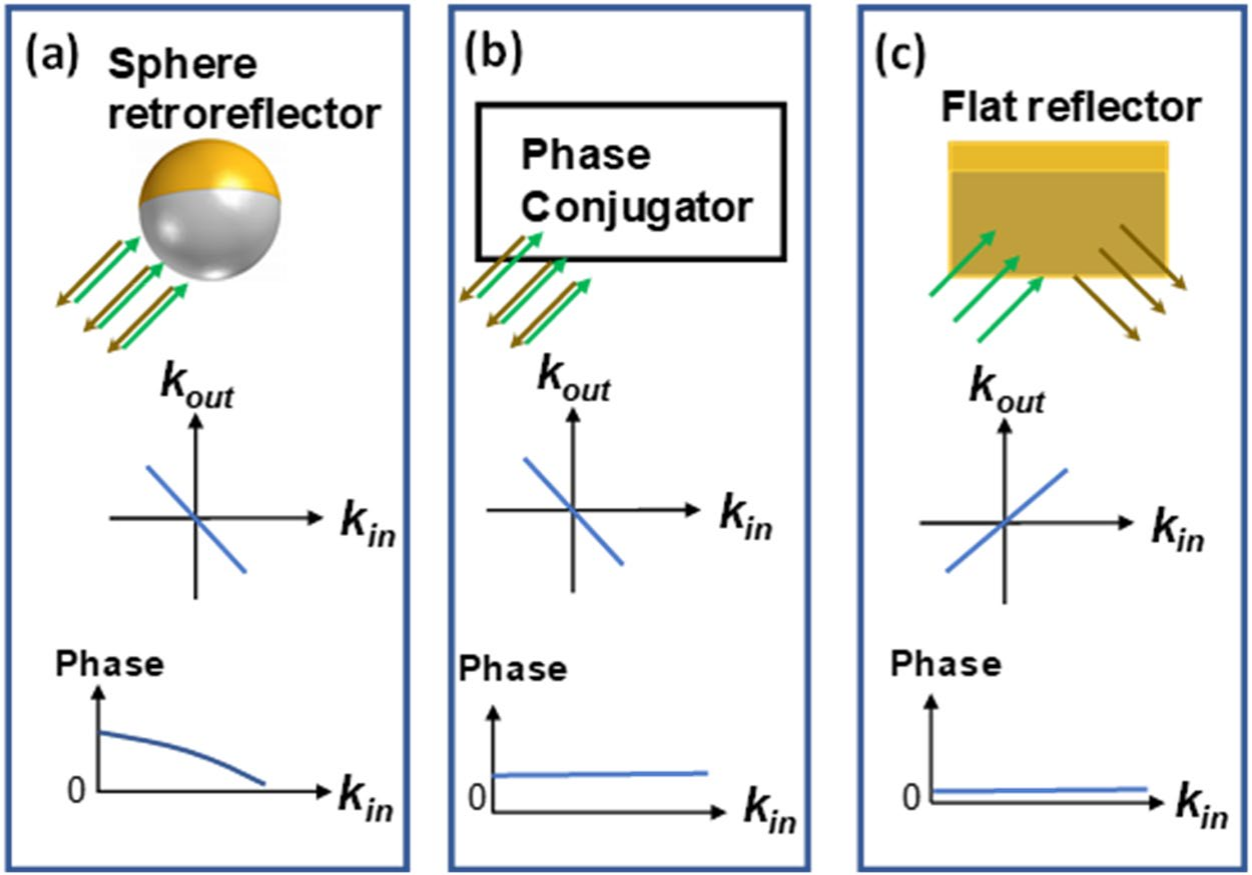
The magnitude and the phase of the output wavevector with respect to the input wavevector. These relations are shown for (**a**) A retroreflector (**b**) A phase conjugator and (**c**) A flat reflector.

Figure 1 shows the input (${k}_{in}$) and output $({k}_{out})$ wavevector relations for the three main classes of optical devices namely microsphere retroreflectors, phase conjugators and reflectors (mirrors), which are the focus of this work. For brevity, here we refer to the lateral wavevector (the component of the wavevector orthogonal to the direction of propagation) simply as wavevector.

Let’s start with a finite-sized retroreflector. By definition, for any given input wavelet hitting a retroreflector ($${e}^{ik.r})$$, the output will be a truncated wavelet (due to the finite size of the retroreflector) that follows the same path in the reverse direction ($${e}^{-ik.r+i{\varphi }_{k}})$$. This definition does not put any constraint on the added phase ($${\varphi }_{k})$$ to the output wavelet. In fact, in all our simulations (presented in the supplemental information) for a sphere retroreflector, we obtained a, $${\varphi }_{k}$$-*k*_*in*_ relation as shown schematically in Fig. 1a. The added phase is the key difference between a retroreflector and a phase conjugator (Fig. 1b) that produces the exact conjugated output, i.e $${e}^{-ik.r}$$. Practical non-linear phase conjugators, however, add a constant phase $${\varphi }_{0}$$ to the output^18^. Since $${\varphi }_{0}$$ is independent of the wavevector, it does not affect the process of “undoing beam distortion” through phase conjugation in the turbid media. In the case of a mirror as shown in Fig. 1c, the wavevector does not change and additionally $${\varphi }_{k}=0$$ when the input/output reference planes are exactly on the surface of the mirror. In all cases, the beam should be focused on the optical device, with a size small enough to avoid the beam truncation.

**Figure 2:**
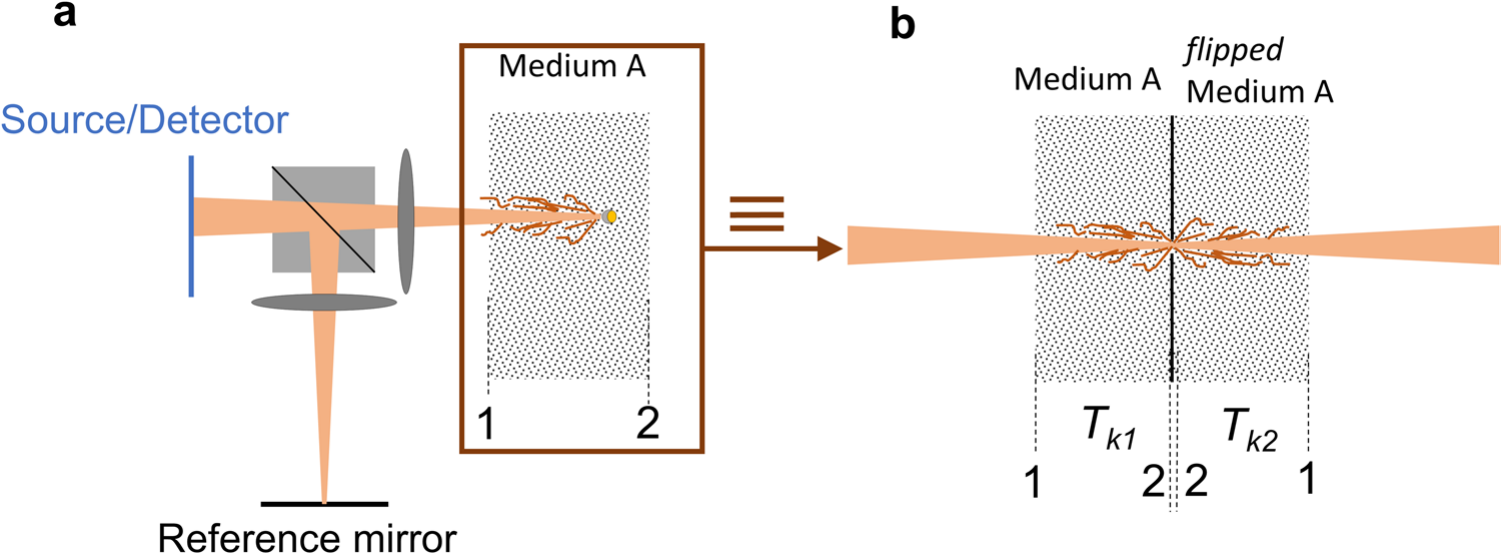
The schematics of a homodyne detection system comprising a reference path and a “sample” path. Combining the beam from these two paths and performing signal processing on the interferogram can reject rays scattered from anywhere else but at the location of the implanted device. The back-reflected light from the optical system of panel (a) can be modeled as *transmission* through an optical system (**b**) that is made of the original turbid medium followed by a flipped version of it across an aperture the size of the retroreflector.

We consider all three optical devices in the context of homodyne detection systems, with the schematics shown in Fig. 2a. Homodyne detection methods have been shown to be very effective in resolving minute reflections within turbid media. Thanks to interferometric effects, these systems can reject the scattered waves with path lengths different than the optical path length of the reference arm. Unfortunately, there is no clear understanding of the potential of homodyne detection of retroreflectors, phase conjugators and the associated time-reversal effects in turbid media. Our mission here is to elucidate the above issues by performing phase sensitive measurements and employing both analytical and numerical evaluations using the TMA method.

It is important to note that the TMA method has been extensively used in the past for a reliable prediction of experimental observations^13,19,20^. To implement this approach, we visualize the system in the transmission equivalent configuration as shown in Fig. 2b. If a retroreflector with finite size is assumed, then the back-reflected light from the optical system can be modeled as transmission through an optical system that is made of the original turbid medium followed by a flipped version of it across an aperture the size of the retroreflector (see Fig. 2b). Based on the properties of the implanted optical device as shown in Fig. 1, we will use the appropriate analytical transmission matrix for this effective aperture. We further use a combination of beam propagation method (BPM) and FDTD full-wave simulations to obtain the transmission matrices numerically. We note that the BPM method is valid when the forward scattering is dominant which is the case for biological issues. We have therefore used BPM to find the transmission matrix of the turbid medium. In contrast to BPM, the FDTD approach does not have this restriction and is suitable for finding the transmission matrix of the reflective optical devices. Knowledge of the transmission matrices enables us to trace the electromagnetic waves without losing the phase information and the coherence effects.

## Results

### Analytical Modeling

The criteria for homodyne coupling are discussed in this section in order to provide an understanding of the experimental observations. The first key factor is that the difference in the optical path length of the rays (denoted by ${d}_{O}$) adversely affects the efficiency of the homodyne detection. The second factor concerns the coupling of the collected light back to the photodetector (denoted by ${\eta }_{C}$). As for the latter, the phase conjugation of the waves ensures that the beam travels along the same path through the post-objective elements to get finally absorbed by the photodetector without experiencing a significant loss. Considering these two factors and under the assumption of normalized illumination power i.e ${\int }^{}{|{E}_{in}(x)|}^{2}dx=\frac{1}{2\pi }{\int }^{}{|{E}_{in}({k}_{x})|}^{2}dx=1$, where *x* and *k*_*x*_ denote the lateral position and wavevector, we define the homodyne detection efficiency as follows:

1 $${\eta }_{h}=\frac{1}{2\pi }\cdot {L}_{p}\cdot {\int }^{}{E}_{in}{|}_{{k}_{x}}\cdot {E}_{out}^{\ast }{|}_{{k}_{x}}d{k}_{x}$$

Where *E*_*in*_ is the incident wave with wavevector of *k*_*x*_ and ${E}_{out}^{\ast }$ is the conjugated scattered wave [originating from *E*_*in*_ *(k*_*x*_)] that reaches the objective lens and its contribution at the same wavevector (*k*_*x*_) is considered in the integrant. *L*_*p*_ is the optical loss of the post-objective system. It is instructive in here to discuss about the above equation and see how it encompasses both discussed factors, i.e *d*_0_ and ${\eta }_{C}$. First, let’s denote the phase difference between ${E}_{in}{|}_{{k}_{x}}$ and ${E}_{out}^{\ast }{|}_{{k}_{x}}$ pair to be *ϕ*(*k*_*x*_). The faster the variation of the $\varphi ({k}_{x})$ with ${k}_{x}$, the larger the reduction in the summation integral of ${\eta }_{h}$ will be. This in fact implies the adverse effect of ${d}_{O}$ on ${\eta }_{h}$. Now, Let’s assume that ${E}_{in}(k)=A{e}^{i{k}_{x}x-i{k}_{z}z}$. For a reflector in the free space, the returned beam will be equal to ${E}_{out}=B{e}^{(i{k}_{x}x-2i{k}_{z}h)}$, where *h* is the distance between the objective lens and the reflector. In contrast to the reflector, a phase conjugator reverses the direction of the wavevector resulting in ${E}_{out}=B{e}^{(-i{k}_{x}x-2i{k}_{z}h)}$. Such a returned beam experiences significantly lower loss before reaching the photodetector and will have the maximum ${\eta }_{c}$. Consequently, we can see at the wavevector of *k*_*x*_, ${E}_{out}^{\ast }\,\,$has a contribution equal to ${B}^{\ast }{e}^{2i{k}_{z}h-\varphi ({k}_{x})}$ for the retroreflector and zero contribution for the case of a mirror. Thus, the description of ${\eta }_{h}\,\,$in Eq. 1 incorporates the effect of coupling (${\eta }_{c})$.

We consider the transmission analog of the reflection measurement as shown in Fig. 2b in order to use the language of TMA for evaluation of the reflected waves. As a result, we could obtain the homodyne detection efficiency of the reflector $({\eta }_{r})$ and the retroreflector $({\eta }_{rr})$ as detailed in the Supplemental information. In particular, their ratio is:

2 $$\frac{{\eta }_{rr}}{{\eta }_{r}}=\frac{8d}{\sqrt{\pi }{L}_{c}}\frac{{{\rm{e}}}^{-{({P}_{1}/{L}_{c})}^{2}}}{{\rm{erf}}[({P}_{1}+2d)/{L}_{c}]-{\rm{erf}}[({P}_{1}-2d)/{L}_{c}]}$$

The coefficients *P*_0_, *P*_*1*,_ and *P*_2_ are the polynomial coefficients describing the added phase to plane waves reaching to the other side of the turbid medium versus their wavevectors, i.e ${\varphi }_{turbid}({k}_{x})={P}_{0}+{P}_{1}{k}_{x}+{P}_{2}{k}_{x}^{2}$. For a homogeneous scattering medium, the effective refractive index is not independent of the wavevector. It rather varies smoothly with the wavevector and the dispersion deviates from that of a non-scattering medium for which *P*_1_ = 0. The phase dispersion of the homogenously scattering medium is the dominant contributor of the beam distortion and is characterized in terms of these polynomial coefficients which depend on the scattering properties of the medium, i.e the scattering mean free path and the anisotropy factor (the average cosine of the scattering angle). In Eq. (2), *d* is the diameter of the device (either reflector or retroreflector) and *L*_*c*_ is the characteristic lateral coherence length for the uniform host medium.

In accordance with our expectation, at two opposite extremes, the ratio of the efficiencies (Eq. 2) reaches unity: 1-For very small reflector/retroreflector, $d\to 0$, which means both devices become essentially point scatterers 2- When $d\to \infty$ (ideal reflector/retroreflector), ${L}_{c}\to \infty$ while ${L}_{c}\gg d$ (which ensures collimation within the reflector/retroreflector). We found the value of the parameters in Eq. 2 and calculated the splitting in the optical losses [10*log*_10_(${\eta }_{rr}$/${\eta }_{r}$)], which is in excellent agreement with our measurements.

### Numerical Simulations

We performed a numerical study for verification of the proposed theory and the experimental observations. We traced both the phase and amplitude of the electromagnetic waves by combining beam propagation and finite difference time domain (FDTD) methods to achieve an accurate and computationally-efficient estimation of the homodyne detection efficiency. We considered the transmission equivalent configuration of the system as shown in Fig. 2b and numerically calculated the relevant transmission matrices to relate the input and output beams in the *k*-space domain. In essence, this method is equivalent to decomposing the input beam to its Fourier components and then tracing each wavelet separately. The reader is referred to Supplemental information for more details.

As mentioned before, there are two key parameters of optical scattering, the mean free path of the scattering and the anisotropy factor. These two parameters are used in the Henyey-Greenstein phase function description of biological tissues^21–23^. Randomly positioned dielectric microspheres in a host material have been widely used for simulation and fabrication of tissue phantoms. While the mean free path is dependent on the concentration of the dielectric spheres, the scattering anisotropy is affected by their radius and the index contrast. Using FDTD simulation we found that microspheres with a diameter of 1.6 μm and an index contrast of 0.03 in a host medium with a refractive index of 1.33 produce anisotropy factor of 0.89. This value is close to the anisotropy factor of a wide variety of tissues including white and grey brain matter which we used in our experiments. The concentration of these microspheres was set to 1.5 × 10^−3^ 1/μm^2^ to yield scattering mean free path of 0.1 mm. We then performed simulations of one dimensional (1D) propagation of the wavefronts in a 2D geometry using beam propagation method to find the transmission matrices for such turbid phantoms.

**Figure 3:**
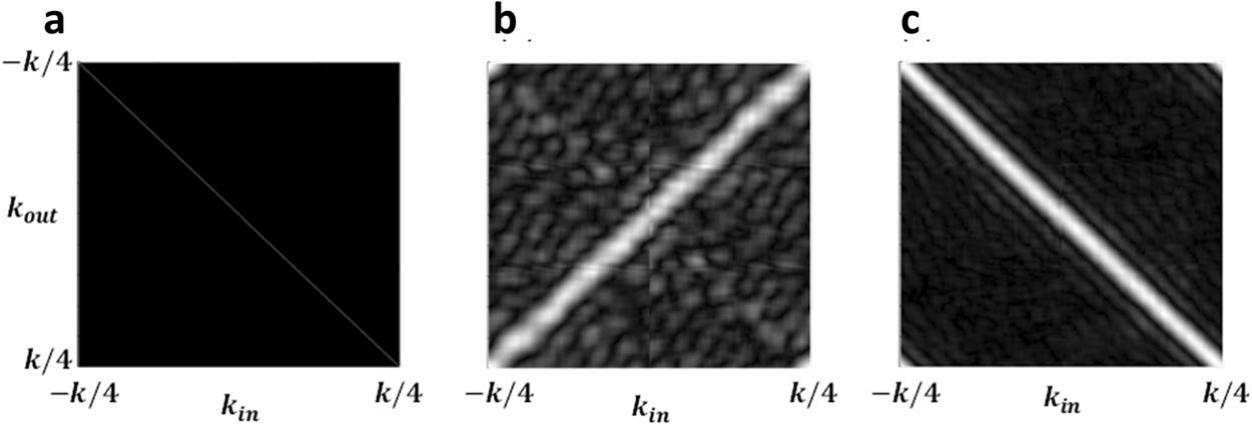
The calculated transmission matrices. (**a**) The turbid medium $({{\boldsymbol{T}}}_{{\boldsymbol{k}},1})$ (**b**) A 50-µm retroreflector (**c**) A flat mirror with a diameter of 50 µm.

Figure 3 shows the calculated transmission matrix for (a) the turbid medium (b) a 50 µm microsphere retroreflector and (c) a 50 µm flat mirror. The amplitude of the transmission matrix of the turbid medium is close to an identity matrix. This result indicates that the change of the in-plane wavevector caused by the propagation of the plane wave within the turbid medium is negligible. The distortion of the output beam is because the scattering affects the phase of the constituent plane waves. In Supplemental information, we formulated this phase distortion.

The calculated transmission matrix of the microsphere retroreflector (Fig. 3b) and the flat reflector (Fig. 3c) are anti-diagonal and diagonal as expected: The retroreflector flips the direction of the wavevector (shown in Fig. 1a) whereas the flat reflector does not affect it (shown in Fig. 1c). The broadening of the transmission matrices is attributed to the finite size of the reflector and the retroreflector and is captured in our formulations in the Supplemental information.

**Figure 4:**
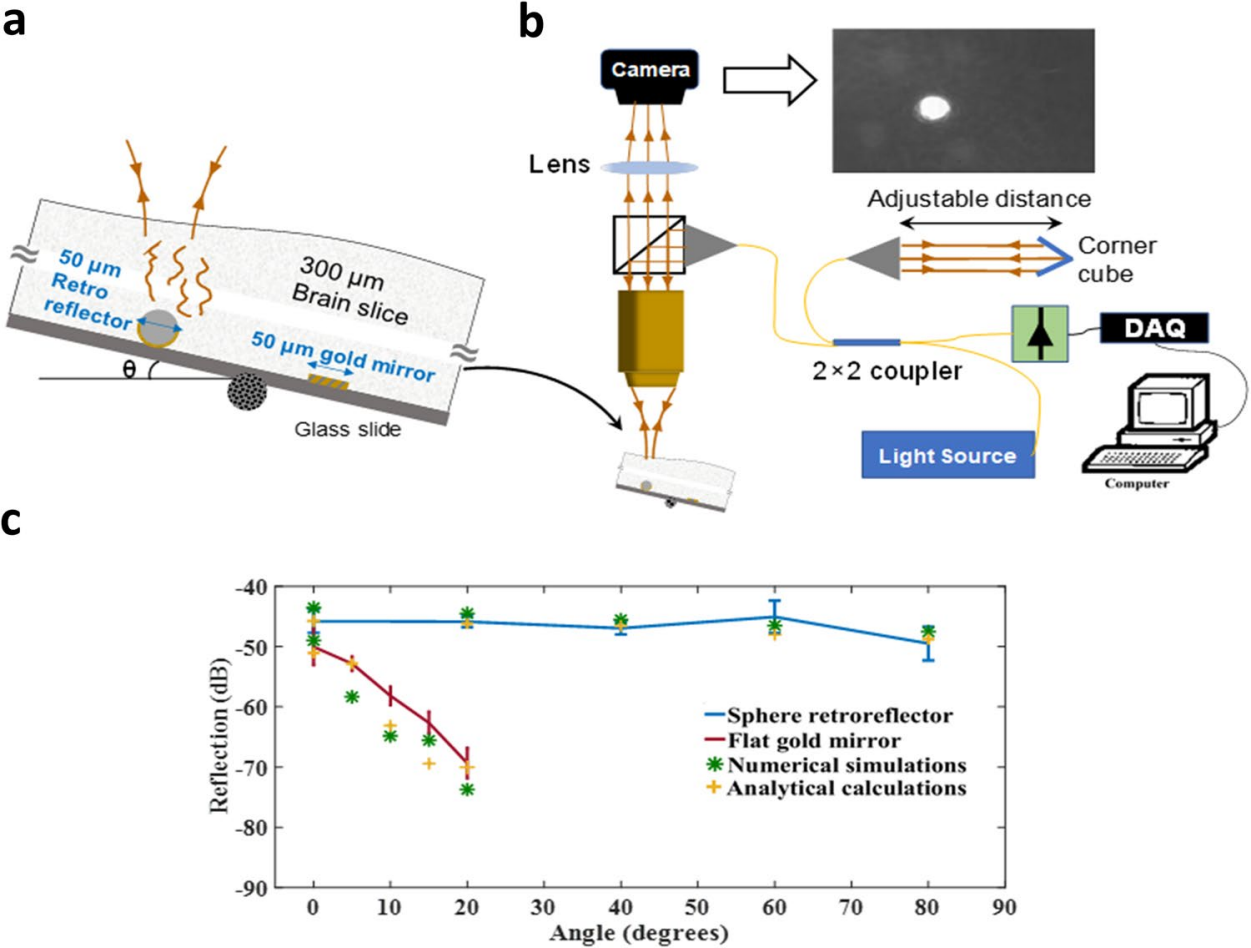
The setup and measurement results. (**a**) Magnified schematics of the experiment, involving a brain slice (as the turbid medium) covering the gold mirror and the microsphere retroreflector. (**b**) The schematics of the homodyne detection system used to measure the reflection versus the sample tilt and (**c**) Homodyne measurement of the reflection for the various tilt of the sample stage. The blue line and error bars show the average and STD of measured reflection for the half-gold coated microspheres with a diameter of 50 µm. The returned signal intensity remains almost flat up to an incident angle of θ~80 degrees. The red line and error bars show the results of the same measurements when the retroreflector is replaced by a flat gold mirror of the same size. Overlaid on these data are the values based on numerical evaluations of the transmission matrices (data points with star markers) and analytical calculations (data points with plus markers). We can see all these values are in good agreement with the measurements.

We used the transmission matrices to calculate the total transmission matrix, i.e ${T}_{k,total}={T}_{k1}{T}_{kr,rr}{T}_{k2}$. We then discretized the input wavevector then multiplied by ${T}_{k,total}\,\,$to obtain the output wavevector ${E}_{out}$. We repeated these calculations for ten different random structures and observed negligible statistical errors (less than 0.2 dB). The numerical evaluation of the homodyne detection efficiencies based on Eq. 1 is in good agreement with both the analytical model and the measurements as shown in Fig. 4.

### Experimental Investigations

In all experiments, a 300-um brain slice covers reflectors and retroreflectors with a diameter of 50 um. Although the well-known corner cube retroreflector (with a size larger than the waist of the input beam) could have been used, it is difficult to fabricate it at the micron scales. On the other hand, half gold coated microspheres are reported in the literature as acting approximately as a good retroreflector^24–26^. It is technically much easier to be made down to submicron dimensions by a single metal evaporation step. Indeed, we have verified the retroreflective property of these objects using full-wave FDTD simulations. The calculated transmission matrix of these devices (Fig. 3b) is similar to that of ideal retroreflectors (Fig. 1a) and consistent with the analytical solution (see Eq. (18) of Supplemental information). Due to the low-cost ease of fabrication and handling, we chose half gold coated microspheres in our experiments. The measurement is repeated ten times with randomly selected reflectors and retroreflectors that were fabricated in parallel, for every angle of incidence by varying the tilt stage angle (*θ*) as shown in Fig. 4a. For each measurement, we scanned our optics in 3D to ensure that the maximum possible back reflected signal was being measured. For these experiments, we prepared a homodyne measurement setup which consists of Agilent reflectometer (8504 A). The Michelson interferometer within this instrument allows highly accurate homodyne measurement of reflection (with a sensitivity of >95 dB). The incident optical power on the brain slice was 1.6 µW at 1550 nm with the measurement bandwidth of 5 Hz. The microscope system has two optical paths separated by a beam splitter and therefore provides two functionalities; to focus the laser on the sample and to obtain magnified video of the surface for laser alignment to the reflector or the retroreflector as shown in Fig. 4b. The measurement results are shown in Fig. 4c which confirms the excellent performance of the half gold coated microspheres as retroreflectors. We can see that the reflection of the flat gold reduces drastically with the tilt while the signal from the retroreflector remains high even under about 80 degrees of rotation. Overlaid on these data are numerical and analytical evaluations. We note that for each data point ten measurements on different devices at various locations were performed to obtain the error bars. Equivalently in our simulations, ten different random media were constructed to evaluate each data point to ensure that the statistical errors are sufficiently smaller than what we observed in practice. The consistency between all these data validates our main assumptions for the homodyne detection of optical scatterers in turbid media and confirms the significant advantage offered by retroreflection and phase conjugation.

## Discussion

In conclusion, we proposed a method to evaluate homodyne detection efficiency of a scatterer inside turbid media and discussed the two critical factors that limit the performance of such systems: the back coupling and the phase coherence among the wavevector spectrum. We derived a generalized analytical relation for the transmission matrix and the homodyne detection efficacy of a reflector (mirror) and a retroreflector. Our method shows good agreement with our experimental results and predicts significant (>5 dB) enhancement of back-reflected light from a retroreflector compared to a reflector at the normal incidence, and many orders of magnitude higher as the incident angle grows. Using the same measurement apparatus, we measured the normal incidence reflection of the retroreflector and the reflector in free-space and confirmed that the difference is less than 1 dB. Therefore, the intrinsic loss cannot justify the observed difference in ${\eta }_{h}$ of the reflector and the retroreflector (>5 dB) in presence of the turbid medium. We show for the first time that the quasi-phase conjugation of the microsphere retroreflector is responsible for this enhancement, producing an effect similar to the time-reversal in a turbid medium. Moreover, the back-reflected signal from microsphere retroreflectors within the turbid medium shows little degradation for tilt angles of up to ±80 degrees. In the free space the tilted flat mirror deflects light and therefore will have a lower homodyne detection efficiency $({\eta }_{h})$ than a tilted retroreflector. Here, we proved that this is still the case in the scattering media where ${\eta }_{h}$ of a tilted flat mirror is significantly lower than that of a retroreflector owing to the time-reversal invariance of light propagation. Our numerical approach and analytical simulations can be applied to a wide variety of the optical communication systems that are based on light scattering and coherent detection.

## Methods

As described in the text, we performed simulations using the beam propagation method (BPM) with Rsoft to obtain the transmission matrix of the medium considering the fact that forward scattering is predominant in biological tissue. Two hundred lateral wavefronts spanning from $-{k}_{0}/4$ to ${k}_{0}/4$ were launched from either side of the scattering medium and fast Fourier transform (FFT) and rearrangement of output yielded the transmission matrix ${T}_{k1,2}$ (the subscript number denotes the input and output sides as shown in Fig. 2 in the text). We payed special attention to the phase of the input wavefronts and made sure that their phases are all zero. The phase of the diagonal elements of ${T}_{k1,2}$ give the dispersion of the scattering medium from which a 3^rd^ order polynomial fitting extracts the *P* coefficients which appear in our analytical formulations.

Replacing the scattering medium in our simulations with the reflector/retroreflector yields the corresponding transmission matrices $({T}_{kr,rr})$. But, for this simulation, we employed the FDTD method (Using Lumerical) rather than BPM to take into account near field and high angle of scattering. Phase matching layer (PML) boundary condition was applied to all boundaries and the mesh accuracy of the simulation region was 10 nm. Similar to the BPM simulations, we performed two hundred simulations per transmission matrix for lateral wavevectors that are evenly distributed within $-{k}_{0}/4$ to ${k}_{0}/4$.

We also used the FDTD method to simulate the scattering properties of the small dielectric spheres which construct the turbid media. In our simulations, a plane wave at the wavelength of 1550 nm illuminates the small dielectrics. A 2D simulation is performed with mesh accuracy of 5 nm in both directions and PML boundary condition was applied to all boundaries. The far-field profile of the scattered field is recorded to find the scattering anisotropy factor.

## Supplementary information

Evaluation of the Returned Electromagnetic Signal from Retro-reflectors in Turbid Media

## References

[1] Hoover EE, Squier JA (2013). Advances in multiphoton microscopy technology. Nature photonics.

[2] Truong TV, Supatto W, Koos DS, Choi JM, Fraser SE (2011). Deep and fast live imaging with two-photon scanned light-sheet microscopy. Nature methods.

[3] Palczewska G (2014). Noninvasive two-photon microscopy imaging of mouse retina and retinal pigment epithelium through the pupil of the eye. Nature medicine.

[4] Zong W (2017). Fast high-resolution miniature two-photon microscopy for brain imaging in freely behaving mice. Nature methods.

[5] Wolf S (2015). Whole-brain functional imaging with two-photon light-sheet microscopy. Nature methods.

[6] Robles FE, Wilson C, Grant G, Wax A (2011). Molecular imaging true-colour spectroscopic optical coherence tomography. Nature photonics.

[7] Liu L (2011). Imaging the subcellular structure of human coronary atherosclerosis using micro–optical coherence tomography. Nature medicine.

[8] Vinekar A (2010). A novel technique using spectral-domain optical coherence tomography (Spectralis, SD-OCT + HRA) to image supine non-anaesthetized infants: utility demonstrated in aggressive posterior retinopathy of prematurity. Eye.

[9] Yaqoob Z, Psaltis D, Feld MS, Yang C (2008). Optical phase conjugation for turbidity suppression in biological samples. Nature photonics.

[10] Hsieh C-L, Pu Y, Grange R, Laporte G, Psaltis D (2010). Imaging through turbid layers by scanning the phase conjugated second harmonic radiation from a nanoparticle. Optics express.

[11] Wang YM, Judkewitz B, DiMarzio CA, Yang C (2012). Deep-tissue focal fluorescence imaging with digitally time-reversed ultrasound-encoded light. Nature communications.

[12] Freund I, Rosenbluh M, Feng S (1988). Memory effects in propagation of optical waves through disordered media. Physical review letters.

[13] Mosk AP, Lagendijk A, Lerosey G, Fink M (2012). Controlling waves in space and time for imaging and focusing in complex media. Nature photonics.

[14] Hillman TR (2013). Digital optical phase conjugation for delivering two-dimensional images through turbid media. Scientific reports.

[15] Ryu J, Jang M, Eom TJ, Yang C, Chung E (2016). Optical phase conjugation assisted scattering lens: variable focusing and 3D patterning. Scientific reports.

[16] Judkewitz B, Wang YM, Horstmeyer R, Mathy A, Yang C (2013). Speckle-scale focusing in the diffusive regime with time reversal of variance-encoded light (TROVE). Nature photonics.

[17] Xu X, Liu H, Wang LV (2011). Time-reversed ultrasonically encoded optical focusing into scattering media. Nature photonics.

[18] Fisher, R. A. *Optical phase conjugation*. (Academic Press, 2012).

[19] Judkewitz B, Horstmeyer R, Vellekoop IM, Papadopoulos IN, Yang C (2015). Translation correlations in anisotropically scattering media. Nature physics.

[20] Kim M (2012). Maximal energy transport through disordered media with the implementation of transmission eigenchannels. Nature photonics.

[21] Hassaninia I, Bostanabad R, Chen W, Mohseni H (2017). Characterization of the optical properties of turbid media by supervised learning of scattering patterns. Scientific reports.

[22] Henyey LG, Greenstein JL (1941). Diffuse radiation in the galaxy. The Astrophysical Journal.

[23] Cornette WM, Shanks JG (1992). Physically reasonable analytic expression for the single-scattering phase function. Applied optics.

[24] Zhang J, Liu J, Wang LM, Li ZY, Yuan Z (2017). Retroreflective‐type Janus microspheres as a novel contrast agent for enhanced optical coherence tomography. Journal of biophotonics.

[25] Bingham W. K. Retroreflective microspheres having a dielectric mirror on a portion of their surface and retroreflective constructions containing such microspheres. United States patent US 3,700,305. 1972 October 24.

[26] Palmquist P. V. & Beck W. R. Ass beads hemispherically reflectorled with metallic coating and compositions thereof. United States patent US 2,963,378. 1960 December 6.

