# Supplementary material for: Evaluation of the Returned Electromagnetic Signal from Retro-reflectors in Turbid Media
Source: Sci Rep. 2019 Apr 25;9:6550. doi: 10.1038/s41598-019-43059-z (PMC6484034; doi:10.1038/s41598-019-43059-z)
Supplement: Supplementary file 1 — Evaluation of the Returned Electromagnetic Signal from Retro-reflectors in Turbid Media [file 41598_2019_43059_MOESM1_ESM.pdf]

Supplementary Information:

Evaluation of the Returned Electromagnetic Signal from Retro-reflectors in Turbid Media

Iman Hassani nia, Skyler Wheaton and Hooman Mohseni

Department of Electrical Engineering and Computer Science, Northwestern University, Evanston, IL 60208 USA

A. Derivation of the Transmission matrix of the System:

The system includes a turbid medium and an implanted reflector/retroreflector as shown in Fig. 2a of the main text. We break up the system in the turbid medium and the reflector/retroreflector. We then combine the corresponding transmission matrices to find the transmission matrix of the whole system. To see the rule for the combination of the transmission matrices, we start by expressing the definition of the transmission matrix[1]:

$$E_2(k_2) = \int T_{12}(k_2, k_1)E_1(k_1)dk_1 \tag{1}$$

Re-applying the above rule, we can find evaluate $E_3(k_2)$ which is the electric field at the third interface:

$$E_3(k_3) = \int T_{23}(k_3, k_2)[\int T_{12}(k_2, k_1)E_1(k_1)dk_1]dk_2 \tag{2}$$

Rearranging the integral yields:

$$E_3(k_3) = \int [\int T_{23}(k_3, k_2)T_{12}(k_2, k_1)dk_2]E_1(k_1)dk_1 \tag{3}$$

which based on the definition of the transmission matrix, as shown in Eq.1, concludes that:

$$T_{13}(k_3, k_1) = \int T_{23}(k_3, k_2)T_{12}(k_2, k_1)dk_2 \tag{4}$$

We will use this combination rule in the next section to derive the transmission matrix of the whole system.

A.1. Transmission matrix of the Turbid medium:

Each wavelet, characterized by the lateral wavevector of k_{in}, propagates inside the turbid medium with negligible coupling to other wavelets with different lateral wavevectors. This has been supported by our numerical simulations in which the wavelet has been inputted to a randomly distributed inhomogeneous medium and the fast fourier transform of the input and output was compared. As shown in Figure 1, the more the scattering, the more the rise of the noise floor will be, but the peak always remains strongly dominant. On the other hand, the wavelet gains a phase, $C(k_{in})$, that is dependent on the effective index of the medium for k_{in}. Therefore, the transmission matrix of the turbid medium in the k-space is represented by a strongly diagonal and unitary matrix with a non-linear phase dispersion as follows:

$$T_{12}(k_{out}, k_{in}) = \delta(k_{out} - k_{in})\exp(-C_{12}(k_{in})t) \tag{5}$$

Corresponding author: **hmohseni@northwestern.edu**
Tel: (847) 491-7108

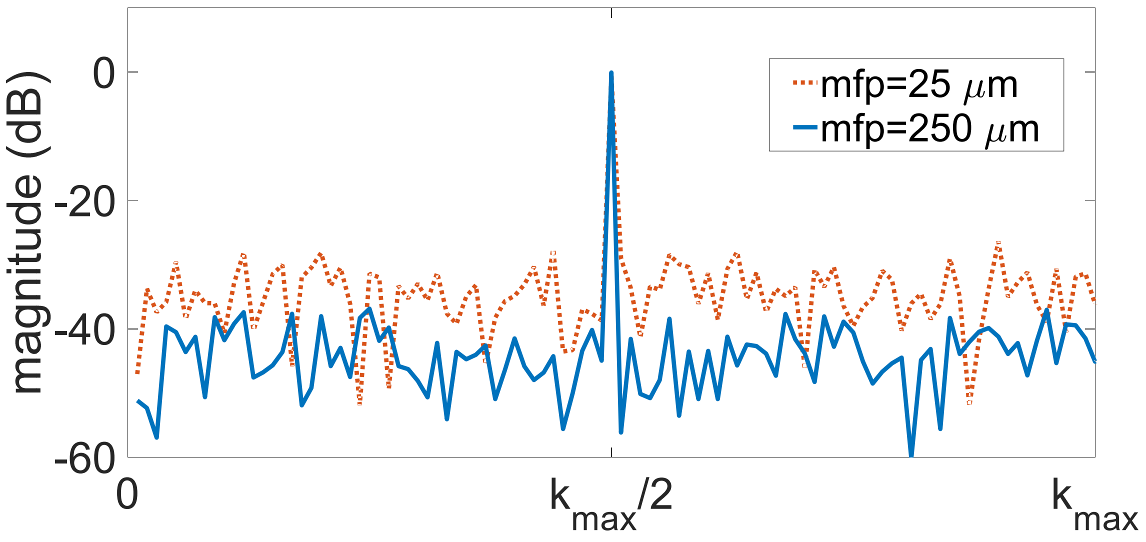

Figure 1. The output spectrum shows that the input wavevector remains dominant, but the noise floor rises for lower mean free path length (mfp).

Where t is the thickness of the turbid medium and $C(k_{in})$ being:

$$C_{12}(k_{in}) = \sqrt{\left[\frac{n_{eff}2\pi}{\lambda_0}\right]^2 - k_{in}^2} \tag{6}$$

where λ_0 is the free space wavelength and n_{eff} is the effective index of the medium for k_{in} . At this point, we find the transmission matrix for the beam traversing the turbid medium and reflecting from reflector, retroreflector or phase conjugator (see the red dashed line in Fig. 2b of the main text). Using the combination rule of Eq.4, we get:

$$T_{1r}(k_r, k_{in}) = \int T_{r/rr}(k_r, k)T_{12}(k, k_{in})dk = T_{r/rr}(k_r, k_{in})\exp(-\,C_{12}(k_{in})) \tag{7}$$

Where $T_{r/rr}$ is the transmission matrix of either reflector (with subscript r) or retroreflector (subscript rr). The reflected beam travels through the turbid medium again and reaches the optical detection system and, the total transmission matrix of the system can be found using again the combination rule:

$$T_{total}(k_{out}, k_{in}) = \int T_{21}(k_{out}, k)T_{1r}(k, k_{in})dk \tag{8}$$

Using equation (5) and knowing that T_{21} has the same expression as T_{12}, we find T_{total} to be:

$$T_{total}(k_{out}, k_{in}) = T_{r/rr}(k_{out}, k_{in})\exp(-i.[C_{21}(k_{out}) + C_{12}(k_{in})]t) \tag{9}$$

A.2. Transmission matrix of the reflector/retroreflector:

Next step toward finding the transmission matrix of the whole system is to find the transmission matrix of the reflector/retroreflector.

We refer to the transmission equivalent of the system as shown in Fig. 2b of the main text wherein the reflector/retroreflector is replaced by an aperture at the location of x_b. As specified in the same figure, the non-prime and prime notations refer to the planes just before (input) and after (output) the aperture respectively.

A.2.1. Finite reflector (mirror):

For each wavelet, the reflected beam has the same lateral wavevector in the transmission configuration but gets truncated due to the finite size of the reflector. Therefore, we can write:

$$E(x_b') = G_r(x_b'). \int E(k_b) e^{ik_b.x_b'} dk_b \tag{10}$$

Where $G_r(x_b')$ defines the characteristic transmission function of the aperture. Knowing that at the input plane $E(k_b) = \int E(x_b) e^{-ik_b.x_b} dx_b$, we obtain:

$$E(x_b') = G_r(x_b'). \int \int E(x_b) e^{-ik_b.x_b}\, e^{ik_b.x_b'} dk_b dx_b \tag{11}$$

Which based on the definition of the transmission matrix, $E(x_b') = \int T_r(x_b, x_b') E(x_b) dx_b$ gives:

$$T_r(x_b, x_b') = G_r(x_b') \int e^{k_b(x_b'-x_b)} dk_b = G_r(x_b') \delta(x_b' - x_b) \tag{12}$$

And $T\big(k_b, k_b'\big)$can be found by performing flipped 2d Fourier transform as follows:[1]

$$T_k\big(k_b, k_b'\big) = \int \int T_x(x_b, x_b')\, e^{-ik_b'.x_b'} e^{ik_b.x_b} d^2x_b' d^2x_b \tag{13}$$

Resulting in:

$$T_{r,k}\big(k_b, k_b'\big) = \int \int G_r(x_b') \delta(x_b - x_b')\, e^{-ik_b'.x_b'} e^{ik_b.x_b} d^2x_b' d^2x_b =$$

$$\int G_r(x_b'). [\int \delta(x_b - x_b')\, e^{-ik_b'.x_b'} e^{ik_b.x_b} d^2x_b]. d^2x_b' = \int G_r(x_b'). e^{-ix_b'.(k_b'-k_b)}\, d^2x_b' \tag{14}$$

Therefore, we can see that $T_k\big(k_b, k_b'\big)$ depends on the difference between k_b' and k_b. So in general, with $\Delta k = k_b' - k_b$, we can write for the aperture:

$$T_{r,k}\big(k_b, k_b'\big) = \mathcal{F}^{k\to\Delta k}\, [\, G_r(x_b')] \tag{15}$$

In the case of the reflector with a finite thickness (t_r), we consider the input and output planes to be split and shifted by $-t_r$ and $+t_r$ away from the central plane b. Assuming that the reflector is a cubic object, the phase added to the propagating wave should be added to the above relation to get the total transmission matrix:

$$T_{r,k}\big(k_b, k_b'\big) = \exp(-i.\, C_{r,21}\big(k_b'\big) t_r) \mathcal{F}^{k\to\Delta k}\, [\, G_r(x_b')] \exp(-C_{r,12}(k_b) t_r) \tag{16}$$

Based on flipped Fourier transform properties, the addition of these phase terms is equivalent to a lateral shift of the emergence of the reflected beam in x space.

A2.2. Finite retroreflector:

For a retroreflector, the direction of the lateral wavevector flips and Eq. (10) will be changed to:

$$E(\acute{x_b}) = \int G_r(\acute{x_b}).E(k_b)e^{-ik_b.\acute{x_b}}dk_b = G_r(\acute{x_b}).\int E(k_b)e^{-ik_b.\acute{x_b}}dk_b \qquad (17)$$

Following the same procedure as the reflector, we obtain the transmission matrix of the retroreflector:

$$T_{rr,k}\left(k_b,\acute{k_b}\right) = \exp(-C_{r,21}\left(\acute{k_b}\right)t_r)\mathcal{F}^{k\to k_b+\acute{k_b}}\left[\,G_r(\acute{x_b})\right]\exp(-C_{r,12}(k_b)t_r) \qquad (18)$$

For abrupt aperture represented by normalized box function G_r, the Fourier transform is Sia nc function. In the limiting case of a very large optical device, the $Sinc$ coefficient approaches the delta-Dirac function forcing the coupling of wave tthe o the opposite wavevector confirming our description of retroreflectors (see Fig. 1a of main text). As mentioned before, the exponential phase terms result in a lateral shift in x space; a deviation from an ideal phase conjugator.

A2.3. Phase conjugator:

According to Fig. 1 of the main text, the main difference between a retroreflector and a phase conjugator is the phase which for the latter is independent of the lateral wavevector. Therefore, we can modify the relation we obtained for the retroreflector to get:

$$T_{pc,k}\left(k_b,\acute{k_b}\right) = \exp(-i\phi_0)\mathcal{F}^{k\to k_b+\acute{k_b}}\left[\,G_r(\acute{x_b})\right] \qquad (19)$$

A.3 The effect of the tilt

A.3.1 Tilt of the reflector

The tilt of the reflector results in the shift of lateral wavevector. Consider a reflector tilted α degrees as shown in Figure 2. The angle of the output wavelet is calculated by adding the tilt of the mirror and the incidence angle. As a result, the magnitude of output lateral wavevector will differ from that of input. For any arbitrary lateral wavevector, with the angle α and β shown in Figure 2, the output lateral wavevector becomes:

$$k_{out} = k_0.\sin(\beta - 2\alpha) = -k.\sin\left(2\alpha - Arcsin\left(\frac{k_{in}}{k}\right)\right) = -\sin(2\alpha)\sqrt{k^2 - k_{in}^2} + \cos(2\alpha)\,k_{in} \qquad (20)$$

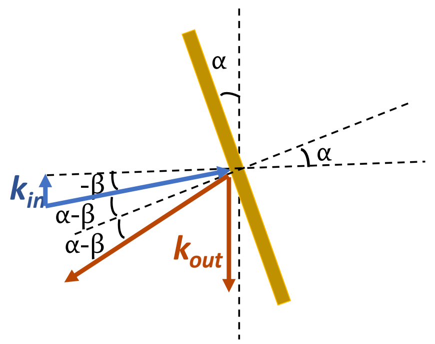

Figure 2 The effect of the tilt of the mirror on the output wavevector.

Another factor that affects the transmission matrix is the change of the apparent size of the mirror (or aperture in the transmission configuration) which is equal to $\acute{d} = d.cos(\alpha)$.

Combining these two effects and using Eq(16), we obtain:

$$T_{r,k}(k_{out}, k_{in}) = d.cos(\alpha).Sinc[d.cos(\alpha)\Bigg(cos(2\alpha)k_{in} - \sin(2\alpha)\sqrt{k_{out}^2 - k_{in}^2} - k_{out}\Bigg)]e^{-i(C_{r,21}(k_{out})+C_{r,12}(k_{in}))t_r} \quad (21)$$

Which is simplified to

$$T_{r,k}(k_{out}, k_{in}) = d.cos(\alpha).Sinc[d.cos(\alpha)(cos(2\alpha)k_{in} - k_{out} - \sin(2\alpha)\,k_0)]e^{-i(C_{r,21}(k_{out})+C_{r,12}(k_{in}))t_r} \quad (22)$$

A3.2 Tilt of the retro-reflector

In this case, the tilt does not change the output lateral wavevector, it rather changes the apparent size of the retroreflector, i.e., $d = d.cos(\alpha)$.Therefore we have:

$$T_{rr,k}(k_{out}, k_{in}) = d.cos(\alpha).Sinc[d.\cos(\alpha)\,(k_{out} + k_{in})]e^{-i(C_{rr,21}(k_{out})+C_{rr,12}(k_{in}))t_{rr}} \quad (23)$$

A3.3 Tilt of the phase conjugator

Starting from Eq.23 and replacing the phase term by a constant phase, ϕ_0, we get:

$$T_{pc,k}(k_{out}, k_{in}) = d.cos(\alpha).Sinc[d.\cos(\alpha)\,(k_{out} + k_{in})]e^{-i\phi_0} \quad (24)$$

A4. Deriving the heterodyne detection efficiency

A4.1 For the implanted flat mirror:

We first find the total transmission matrix, $T_{total}(k_{out}, k_{in})$, by combining Eq. 22 and Eq.9:

$$T_{total}(k_{out}, k_{in}) = d.cos(\alpha).Sinc[d.\cos(\alpha)(cos(2\alpha)k_{in} - k_{out} - \sin(2\alpha)\,k_0)] \times e^{-i(C_{r,21}(k_{out})+C_{r,12}(k_{in}))t_r - i.[C_{21}(k_{out})+C_{12}(k_{in})]t} \quad (25)$$

We can then use the transmission analog of the reflection measurement (shown in Fig. 2b of the main text)to apply the TMA method for evaluation of the reflected waves:

$$E_{out}(k_{out}) = T_{k,total}(k_{out}, k_{in})E_{in}(k_{in})\Delta k_h \quad (26)$$

Where Δk_h is the wavevector resolutions of the system limited by the coherence length of the light source.

Subsequently, we find the output with wavevector, $k_{out} = -k$, when the incident wavelet has a wavevector of $k_{in} = k$:

$$E_{out}(-k) = dcos(\alpha).Sinc[dcos(\alpha)((1 + \cos(2\alpha))\,k - \sin(2\alpha)\,k_0)]e^{-i(P_0+P_1k+P_2k^2)}E_{in}(k)\Delta k_{char} \quad (27)$$

where we have also done a 3rd order polynomial fitting to the phase, i.e $\left(C_{21}(-k)+C_{12}(k)\right)t+\left(C_{r,21}(-k)+C_{r,12}\,(k)\right)t_r=P_0+P_1k+P_2k^2$. The illuminating Gaussian wave at the input plane (the top surface of the tissue) is described by $E_{in}(k_x)=\left(\frac{2\pi a_1}{a_1^2+a_2^2}\right)^{1/4}\exp(-\frac{1}{4}\frac{k_x^2}{a_1+a_2i})$. The coefficient a_1 is equal to r_0^{-2} with r_0 being the radius of the beam on the plane of illumination. Parameter a_2 does not have a direct physical interoperation, but together with parameter a_1 determines the numerical aperture of the incident beam; $NA=1/\sqrt{1+\frac{1}{4}\frac{(a_1a_2^2k_0^2)}{(a_1^2+a_2^2)^2}}$. Performing the integration for finding the homodyne detection efficiency based on Eq.1 of the main text, yields:

$$\eta_r=\frac{1}{2\pi}.L_p\times 2\left(\frac{2\pi a_1}{a_1^2+a_2^2}\right)^{0.5}dcos(\alpha)\Delta k_{char}\int_0^\infty Sinc(dcos(\alpha)((1+\cos(2\alpha))\,k-\sin(2\alpha)\,k_0))\exp(-i(P_0+P_1k+P_2k^2))\exp(-\frac{1}{2}\frac{k^2}{a_1+a_2i})dk \tag{28}$$

Which by introducing the variable β becomes equal to:

$$\eta_r=A\int_0^\infty\int_0^{dcos(\alpha)(1+\cos(2\alpha))}cos(\beta(k-B)\exp\left(-i(P_0+P_1k+P_2k^2)\right)\exp\left(-\frac{1}{2}\frac{k^2}{a_1+a_2i}\right)d\beta dk \tag{29}$$

In here, we define two variables, A, and B to simplify the above equation:

$$A=\frac{1}{2\pi}.L_p\times 2\left(\frac{2\pi a_1}{a_1^2+a_2^2}\right)^{0.5}\Delta k_{char}(1/1+\cos(2\alpha)]) \tag{30a}$$

$$B=\frac{\sin(2\alpha)k_0}{1+\cos(2\alpha)} \tag{30b}$$

In order to solve Eq.29, we expand the cosine term:

$$\eta_r=A/2\int_0^\infty\int_0^{dcos(\alpha)(1+\cos(2\alpha))}[\exp\left(-i((P_0-\beta B)+(P_1+\beta)k+P_2k^2)\right)+\exp\left(-i((P_0+\beta B)+(P_1-\beta)k+P_2k^2)\right)]\exp\left(-\frac{1}{2}\frac{k^2}{a_1+a_2i}\right)d\beta dk \tag{31}$$

Rearranging the terms in the exponent, we get for the first term:

$$\int_0^\infty exp(-\frac{L_c^2}{4}\left(k-\frac{i(P_1+\beta)}{\frac{1}{2}L_c^2}\right)^2+i\beta B-(P_1+\beta)^2/L_c^2)dk \tag{32}$$

Which we have neglected the constant phase term P_0. The second term in the integral can be found similarly by replacing β with $-\beta$. These expressions are easily tractable with gaussian integral rules. By integrating over k , we can simlify the equation for the efficiency as follows:

$$\eta_r=\frac{A}{2}\sqrt{\pi}/L_c\int_0^{dcos(\alpha)(1+\cos(2\alpha))}e^{i\beta B-\frac{(P_1+\beta)^2}{L_c^2}}+e^{-i\beta B-\frac{(P_1-\beta)^2}{L_c^2}}d\beta \tag{33}$$

When the rotation is zero, i.e. $\alpha=B=0$ we obtain:

$$\eta_r=\frac{A}{2}\sqrt{\pi}/L_c\int_0^{2d}e^{-\frac{(P_1+\beta)^2}{L_c^2}}+e^{-\frac{(P_1-\beta)^2}{L_c^2}}d\beta \tag{34}$$

By changing the variable in the first term as $t_1=(\beta+P_1)/L_c$ and for the second term as $t_2=(-\beta+P_1)/L_c$ and using the definition of the $erfc$ function we obtain:

$$\eta_r=A(\frac{\pi}{4})[\mathrm{erf}\left(\frac{P_1+2d}{L_c}\right)-\mathrm{erf}\left(\frac{P_1-2d}{L_c}\right)] \tag{35}$$

This resembles the denominator of Eq.2 of the main text. In general, for non zero angles we get:

$$\eta_r = A(\frac{\pi}{4})\, e^{iBP_1 - \frac{B^2L_c^2}{4}} \int_0^{dcos(\alpha)(1+\cos(2\alpha))} e^{\frac{(P_1 - iBL_c^2/2+\beta)^2}{L_c^2}} + e^{\frac{(P_1 - iBL_c^2/2-\beta)^2}{L_c^2}} d\beta \tag{36}$$

By conducting this integral and neglecting the e^{iBP_1} term we obtain:

$$\eta_r = A(\frac{\pi}{4})\, e^{-\frac{B^2L_c^2}{4}}[\text{erf}\left(\frac{P_1 + dcos(\alpha)(1+\cos(2\alpha))}{L_c} - iBL_c/2\right) - \text{erf}\left(\frac{P_1 - dcos(\alpha)(1+\cos(2\alpha))}{L_c} - iBL_c/2\right)] \tag{37}$$

Which for $\alpha = 0$ reduces to Eq. 35.

A4.2 For the implanted retroreflector:

We employ a similar methodology that we used for the flat mirror in this case. Based on the transmission matrix of the retroreflector (Eq. 23) we obtain:

$$E_{out}(-k) = dcos(\alpha).\,\mathrm{e}^{-i(P_0+P_1k+P_2k^2)}E_{in}(k)\Delta k_{char} \tag{38}$$

Therefore, we will have:

$$\eta_{rr} = A.\,dcos(\alpha)(1+\cos(2\alpha))(\frac{\sqrt{\pi}}{L_c})e^{-\frac{P_1^2}{L_c^2}} \tag{39}$$

The ratio of the homodyne detection efficiency of the retroreflector to that of a mirror can found by dividing Eq.37 by Eq.39:

$$\frac{\eta_{rr}}{\eta_r} = \frac{4dcos(\alpha)(1+\cos(2\alpha))e^{-\frac{P_1^2}{L_c^2}}}{\sqrt{\pi}L_c e^{-\frac{B^2L_c^2}{4}}\left[\text{erf}\left(\frac{P_1+dcos(\alpha)(1+\cos(2\alpha))}{L_c} - \frac{iBL_c}{2}\right) - \text{erf}\left(\frac{P_1-dcos(\alpha)(1+\cos(2\alpha))}{L_c} - \frac{iBL_c}{2}\right)\right]} \tag{40}$$

Which for $\alpha = 0$ reduces to Eq.2 of the main text. We used this formula with a_1=0.007 µm^{-2}, a_2=0.01 µm^{-2}, P_0=3.03, P_1= 0.7380 µm and P_2= -59.7180 µm^2 to fit to experimnental results (data points with plus marker) shown in Fig. 4c of the main manuscipt.

A5. Numerical simulations:

A5.1 Transmission matrix of the turbid medium

The turbid medium is modeled by a set of randomly positioned spheres in a host medium. In the case of brain matter, the host medium has a refractive index of 1.33. Using this value, we performed an FDTD simulation and varied the size of spheres to match the scattering properties of the brain. The anisotropy is characterized by the average cosine of scattering angle (g). The FDTD calculation of the far-field, excluding the source, showed us that the index difference of 0.03 and the sphere size of 1.3 µm results in g of 0.89 close to that of brain matter.

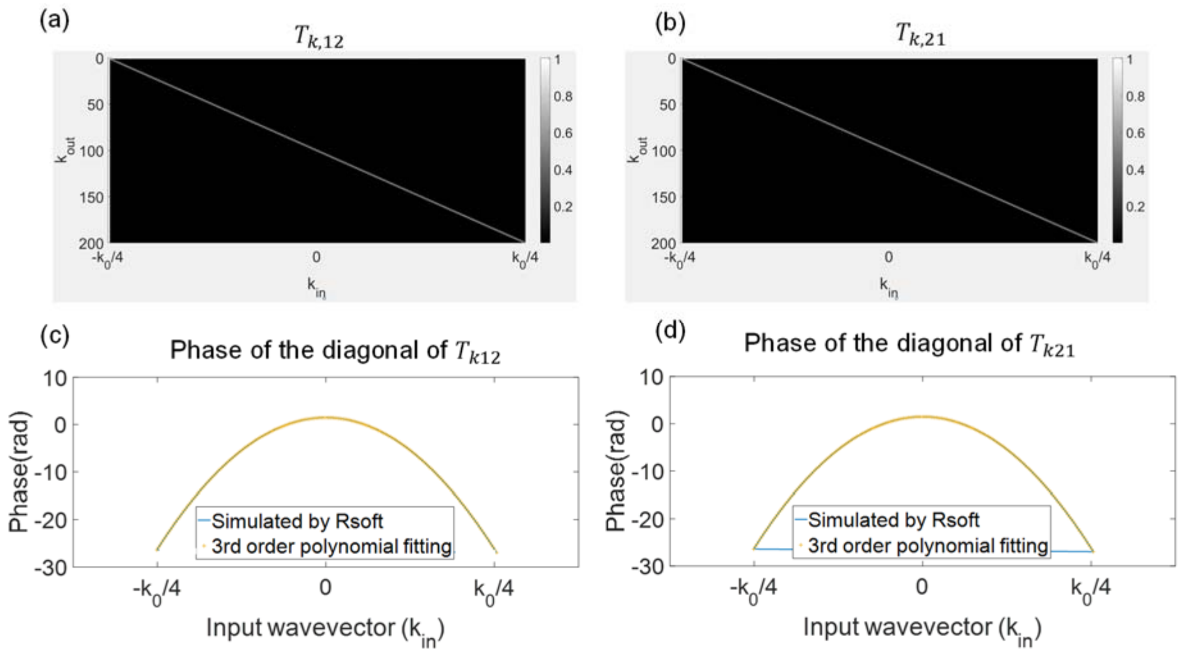

Figure 3 The calculated amplitude of the transmission matrix for **(a)** T_{k12} **(b)** T_{k21} and the phase along the diagonal of **(c)** T_{k12} **(d)** T_{k21}. As can be seen T_{k12} and T_{k21} are almost identical. The reason behind the amplitude of transmission matrices being almost like identity matrices is the fact that in k-space the scattering induces very week and homogenous coupling between k states. In here $T_{k12} \times T_{k21}^{+} = I$ in agreement with the energy conservation rule.

We used the beam propagation method using commercial software (Rsoft) and we built ten different random structures with the same sphere size, concentration and refractive index to gain statistical error bars. We note that the beam propagation method is valid because the scattering within the brain is forward-dominated (g~0.9). The input and output planes were divided into 200 segments with the resolution of 2λ and the wavelets $e^{jk_x x}, \frac{-k_0}{4} < k_x < \frac{k_0}{4}$ were contsructed over the discretizied input plane. We took the FFT of the output (with the same spatial resolution) and then switched the input and output planes and repeated the same procedure to be find $T_{k,12}$ and $T_{k,21}$ respectively. As shown in Fig. a and b , these matrices look similar and they are, almost like an identity matrix except that the phase varies in a nonlinear fashion as shown in Figure 3c and d. Furthermore we verfied , that, $T_{k,12} \times T_{k,21}^{+} = I$ which is consistent with the energy conservation rule since the abosrption of the turbid media is neglected in our Rsoft simulations.

A5.2. Transmission matrix of the reflector/retroreflector

For this simulation, we used full wave FDTD simulations using Lumerical with the simulation setup shown in Figure 4 (a) and (b). We have used phase-matching layers (PML) on all boundaries. The source in here is an import source with values of $k_x$being varied between -k_0/4 to k_0/4. The far-field pattern of a monitor that is located slightly behind the source was used to find the transmission matrices. The simulated transmission matrices are shown in Figure 4 (c) and (d) which are consistent with our theoretical formulas (Eqs.22 and 23).

A5.3. Total transmission matrix

By multiplying the transmission matrices of the turbid medium and the one corresponding to the reflector/retroreflector, we found the transmission matrix of the whole system using the relation:

$$T_{total} = T_{21} T_{r/rr} T_{12} \tag{41}$$

Using these simulations, we found the total transmission matrix and performed the 3rd order polynomial fitting to the phase of the diagonal (anti-diagonal) components of the total transmission matrix of the reflector (retroreflector). With a_1=0.007 µm^{-2}, a_2=0.01 µm^{-2}, P_0=3.03, P_1= 0.7380 µm and P_2= -59.7180 µm^2 and using Eqs 37 and 39, we get the values with star markers as shown in Figure4(c) of the manuscript.

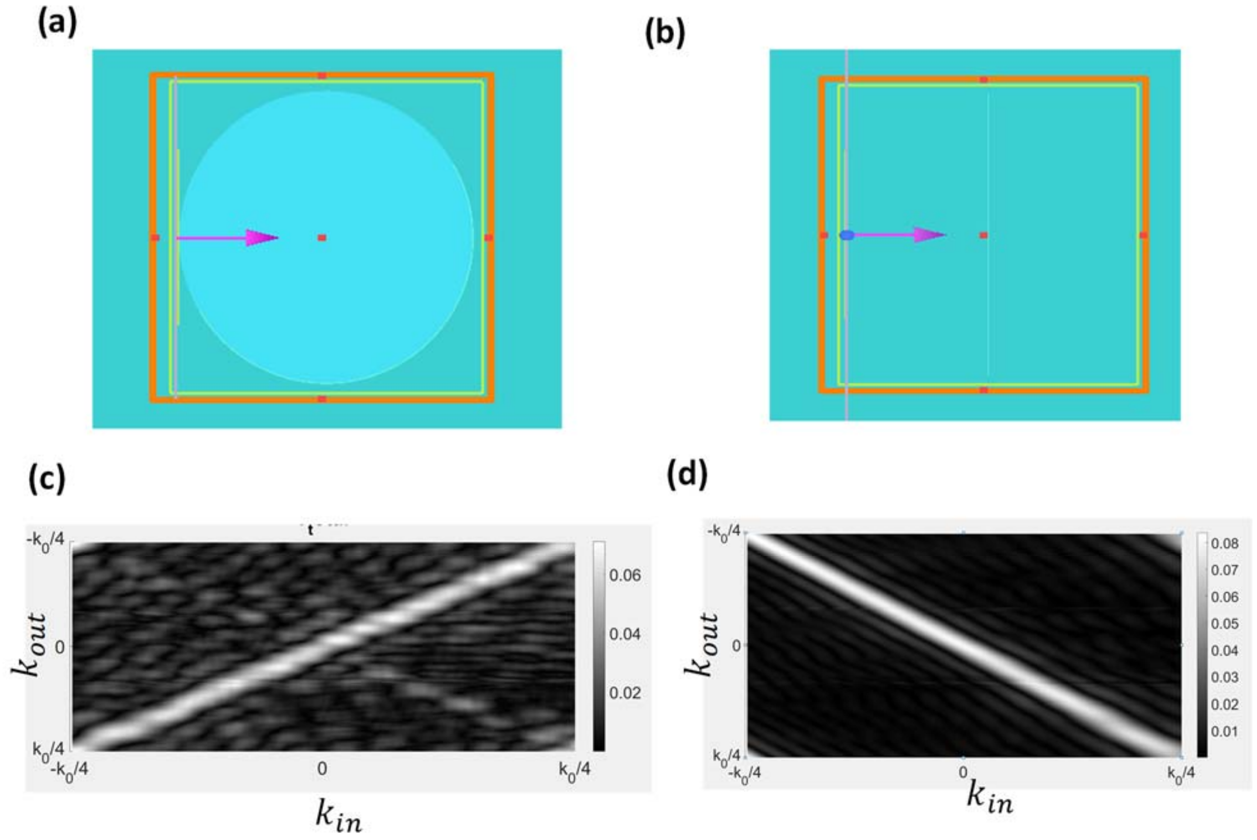

Figure 4 FDTD evaluation of the transmission matrices. The simulation setup for **(a)** a microsphere retroreflector **(b)** A flat mirror. **(c)** the amplitude of the transmission matrix for a retroreflector and (d) the amplitude of the transmission matrix for a flat mirror.

References:

1 Judkewitz B, Horstmeyer R, Vellekoop IM, Papadopoulos IN, Yang C. Translation correlations in anisotropically scattering media. *Nature physics* 2015; **11**: 684.
